# Intrinsic and Antipsychotic Drug-Induced Metabolic Dysfunction in Schizophrenia

**DOI:** 10.3389/fnins.2017.00432

**Published:** 2017-07-28

**Authors:** Zachary Freyberg, Despoina Aslanoglou, Ripal Shah, Jacob S. Ballon

**Affiliations:** ^1^Department of Psychiatry, University of Pittsburgh Pittsburgh, PA, United States; ^2^Department of Cell Biology, University of Pittsburgh Pittsburgh, PA, United States; ^3^Department of Psychiatry and Behavioral Sciences, Stanford University Stanford, CA, United States

**Keywords:** schizophrenia, antipsychotic drugs, metabolism, insulin, diabetes, dyslipidemia, dopamine, monoamines

## Abstract

For decades, there have been observations demonstrating significant metabolic disturbances in people with schizophrenia including clinically relevant weight gain, hypertension, and disturbances in glucose and lipid homeostasis. Many of these findings pre-date the use of antipsychotic drugs (APDs) which on their own are also strongly associated with metabolic side effects. The combination of APD-induced metabolic changes and common adverse environmental factors associated with schizophrenia have made it difficult to determine the specific contributions of each to the overall metabolic picture. Data from drug-naïve patients, both from the pre-APD era and more recently, suggest that there may be an intrinsic metabolic risk associated with schizophrenia. Nevertheless, these findings remain controversial due to significant clinical variability in both psychiatric and metabolic symptoms throughout patients' disease courses. Here, we provide an extensive review of classic and more recent literature describing the metabolic phenotype associated with schizophrenia. We also suggest potential mechanistic links between signaling pathways associated with schizophrenia and metabolic dysfunction. We propose that, beyond its symptomatology in the central nervous system, schizophrenia is also characterized by pathophysiology in other organ systems directly related to metabolic control.

Schizophrenia is a chronic psychiatric illness characterized by hallucinations, delusions, cognitive symptoms, and negative symptoms. Use of antipsychotic drugs (APDs) has been a mainstay of treatment. Though these medications effectively treat the hallucinations, APDs also cause significant metabolic side effects including insulin resistance (IR), dyslipidemia obesity and ultimately type II diabetes (T2D; Ballon et al., 2014). Consequently, a longstanding controversy in managing patients with schizophrenia has been the premorbid risk for adverse metabolic states. We therefore pose the question: is there an intrinsic metabolic risk inherent to schizophrenia or is the metabolic phenotype primarily driven by the impact of APDs?

## Pre-antipsychotic era

Prior to the APD era, cohort studies (Kooy, 1919; Kasanin, 1926) noted increased incidence of abnormal glucose metabolism in people with schizophrenia (Henneman et al., 1954). These observations corroborated cross-sectional results demonstrating that the prevalence of diabetes was greater in patients with schizophrenia compared to the general population (Kasanin, 1926). Examination of the data in pre-APD studies of schizophrenia patients suggests that abnormal glucose metabolism far preceded the confounding effects of pharmacologic intervention. Interestingly, a study in 1922 determined that increased fasting and post-prandial blood glucose levels in untreated patients with schizophrenia was in part correlated to the severity of their illness. Catatonic patients experienced more than twice the maximum post-prandial plasma levels of glucose than disorganized schizophrenia patients. Moreover, the fasting glucose level after a 12-h fast was uniformly higher in catatonic patients than in those with other forms of schizophrenia (Lorenz, 1922).

Yet, there is a wide heterogeneity among the metabolic sequelae associated with schizophrenia across all stages of the illness. In the literature preceding the era of APD treatment, Kasanin (1926) reported on a case series of 154 people with dementia praecox (later renamed schizophrenia), stating: “From this compilation it is evident that we could find no curve that is characteristic of the “dementia praecox” group as a whole. This is not unexpected in view of the heterogeneous character of the clinical conditions described under this head” (Kasanin, 1926). Further, even for those taking medications with the highest metabolic risk such as, olanzapine and clozapine, there is a notable percentage, more than 15%, that do not gain weight (Leadbetter et al., 1992). Rather, these medications may still produce significant metabolic disturbances including hyperglycemia and dyslipidemia that are independent of effects on body mass (Kang and Lee, 2015). While such wide-ranging effects make the study of a definitive mechanism more challenging, they nevertheless demonstrate the likely existence of a metabolic phenotype intrinsic to schizophrenia. Moreover, there is accumulating evidence suggesting the likelihood of multiple pathways responsible for the diversity of metabolic phenotypes in people with schizophrenia.

## Contemporary evidence of intrinsic metabolic risk in schizophrenia

More recent studies have confirmed the pre-medication era findings, and have continued to show impaired glucose tolerance, dyslipidemia, and related aspects of metabolic syndrome in drug-naïve patients with schizophrenia, as compared to healthy controls (Fernandez-Egea et al., 2009; Enez Darcin et al., 2015). In a 2016 case-control study of drug-naïve patients with schizophrenia, almost one-fourth of patients had baseline impaired glucose tolerance compared to none of the controls. The patients with schizophrenia also had higher fasting glucose levels and greater IR. However, compared to the controls, the patients with schizophrenia had higher BMIs, LDL levels, TG levels, waist circumference, and hip circumference. It is unclear to what extent those factors confounded the impaired glucose tolerance observed in patients with schizophrenia, though it is notable that these anthropometric parameters differed between cases and controls before the opportunity for pharmacologic intervention (Chen et al., 2016). Similar findings were found in youth through the recent Tolerability and Efficacy of Antipsychotics (TEA) trial. Drug-naïve first-episode psychosis patients ages 12–17 years had higher waist circumference, total cholesterol, LDL, and non-HDL levels compared to controls (Jensen et al., 2017). These suggestions have been challenged in meta-analyses that exclude patients with affective psychosis (which could lead to confounding changes in dietary or exercise habits). One meta-analysis found that the difference in LDL levels between first-episode, non-affective psychosis patients and controls was clinically insignificant. The systemic review did, however, find that first-episode, non-affective psychosis patients had higher triglyceride levels, and lower HDL levels than the controls. The study suggests that this may represent subclinical dyslipidemia, and hypothesizes that psychosis-specific mechanisms may be at play, perhaps by sharing overlapping genetic regions with metabolic phenotypes (Misiak et al., 2017).

Another case-control of drug-naïve, first-episode patients with psychosis showed higher insulin and C-peptide levels and lower HDL levels compared to the age- and BMI-matched controls. This suggests that even when accounting for the possible effect that schizophrenia itself may have on BMI, IR is still increased in patients with psychosis compared to healthy controls. Interestingly, when controlling for BMI, patients and controls were no different in their triglyceride, total cholesterol, and fasting glucose levels (Petrikis et al., 2015). Indeed, use of APDs certainly increases the risk of metabolic disturbance, but as discussed, there is a baseline elevated risk for metabolic syndrome in people with schizophrenia prior to antipsychotic use. Consistent with this, a meta-analysis of 48 studies showed a 10.2% prevalence of metabolic syndrome in antipsychotic-naïve patients, but a rate of 19.4% for patients on aripiprazole, and 47.2% for patients on clozapine. This further suggests that the disease state has an independent influence on adverse metabolic symptoms beyond the treatment-induced effect (Vancampfort et al., 2015; see Table 1).

**Table 1:** Intrinsic metabolic risk in untreated patients with schizophrenia, amplified by use of antipsychotic drugs, as observed in multiple studies and meta-analyses.

**Year**	**Author**	**Study title**	**Study design**	**Size**	**Notes**
1922	Lorenz	Sugar tolerance in dementia praecox and other mental disorders	Prospective case series	107	Catatonic patients had higher fasting and post-prandial glucose levels compared to patients with other forms of schizophrenia, in the absence of psychotropic drug intervention
2011	Falissard et al.	The METEOR study: frequency of metabolic disorders in patients with schizophrenia. Focus on first and second generation and level of risk of antipsychotic drugs	Cross-sectional	2,270	There were few significant differences between patients prescribed first- or second-generation antipsychotic drugs, in terms of the prevalence of glycemic disorders, dyslipidemia, and metabolic disorder. However, there were higher rates of hypertension in those using first-generation antipsychotics
2015	Petrikis et al.	Parameters of glucose and lipid metabolism at the fasted state in drug-naïve first-episode patients with psychosis: evidence for insulin resistance	Case-control	80	When matching cases and controls by age and BMI, insulin and C- peptide levels remained higher in drug naïve first-episode patients compared to controls, while HDL levels were lower
2015	Vancampfort et al.	Risk of metabolic syndrome and its components in people with schizophrenia and related psychotic disorders, bipolar disorder and major depressive disorder: a systematic review and meta-analysis	Meta-analysis	52,678 (in 198 studies)	Antipsychotic drug-naïve patients had a 10.2% prevalence of metabolic syndrome, compared to 19.4% for patients on aripiprazole and 47.2% for patients treated with clozapine
2016	Chen et al.	Impaired glucose tolerance in first-episode drug-naïve patients with schizophrenia: relationships with clinical phenotypes and cognitive deficits	Case-control	175	Drug-naïve first-episode patients with schizophrenia had higher BMIs, LDL levels, TG levels, waist and hip circumference, compared to controls; one-fourth of drug-naïve patients had impaired glucose tolerance before medication trials
2017	Jensen et al.	Pretreatment Cardiometabolic status in youth with early-onset psychosis: baseline results from the TEA Trial	Case-control	113	Young patients with first-episode psychosis who were drug naïve, ages 12–17, also showed higher waist circumference, total cholesterol, LDL, and non-HDL levels than controls
2017	Misiak et al.	Lipid profile disturbances in antipsychotic-naïve patients with first-episode non-affective psychosis: a systematic review and meta-analysis	Meta-analysis	1,803 (in 19 studies)	First-episode, non-affective psychosis patients had higher triglyceride and lower HDL levels than controls, but LDL levels were similar. A subclinical dyslipidemia could suggest overlapping genetic regions between cardio-metabolic factors and schizophrenia
2017	Pillinger et al.	Impaired glucose homeostasis in first-episode Schizophrenia: a systematic review and meta-analysis	Meta-analysis	1,345 (in 16 studies)	Patients with schizophrenia were at an increased risk of diabetes from the onset of disease, which is only worsened by the effects of chronic illness and long-term use of antipsychotic drugs
2017	Rajkumar et al.	Endogenous and antipsychotic-related risks for diabetes mellitus in young people with Schizophrenia: a Danish population-based cohort study	Ecologic prospective cohort	8,945	Patients with schizophrenia not yet trialed on an antipsychotic drug were three times more likely to develop diabetes than the general population. Risk was further increased three-fold upon starting first- or second-generation antipsychotic drugs, compared to subjects who remained drug-naïve

In addition to individual smaller studies, two large meta-analyses of studies of first-episode and drug-naïve schizophrenia patients further showed a baseline increased risk for metabolic syndrome at the onset of treatment, which only worsened over progression of the illness (Mitchell et al., 2013). Glucose homeostasis was impaired from the onset of schizophrenia, despite patients having similar hemoglobin A1c levels relative to controls. The factors that were higher in patients included fasting plasma glucose levels, glucose levels after an oral glucose tolerance test, fasting insulin levels, and IR. The meta-analysis concluded that patients with schizophrenia were at an increased risk of diabetes, only exacerbated by the effects of chronic illness and long-term treatment (Pillinger et al., 2017).

A population-based study in Denmark tested the added risk of starting APDs. While APD-naïve patients with schizophrenia had a higher rate of diabetes by a factor of 3.07 compared with the general population, the risk of diabetes was 3.64 times higher in patients started on APDs compared to patients with schizophrenia who remained APD-naïve. Both first-generation and second-generation APDs increased this risk more than three-fold, with no statistically significant between the two drug classes in their capacity to cause these metabolic sequelae (Rajkumar et al., 2017).

Impaired glucose tolerance has also been demonstrated in non-psychotic, first-degree relatives of schizophrenia patients, further indicating a heritable phenotype that tracks with risk of psychosis, but is independent of the actual development of a psychotic disorder (Spelman et al., 2007). This lends credence to the concept of a novel metabolic endophenotype associated with schizophrenia with variable penetrance across both affected individuals and their unaffected relatives.

Significantly, the risk of metabolic abnormalities further increases significantly with duration of illness. Chronically-ill subjects exhibit increased rates of metabolic dysfunction compared to first-episode and drug-naïve patients (Mitchell et al., 2013; Correll et al., 2014). Additional lifestyle factors including sedentary lifestyle, poor diet, and increased smoking further add to the metabolic risks associated with schizophrenia (Brown et al., 1999).

## Contribution of antipsychotic medications to metabolic risk

In addition to the intrinsic risk factors for metabolic disease associated with schizophrenia including genetics and lifestyle (Ballon et al., 2014; Heald et al., 2017; Rado, 2017), there is a consensus that APDs can further exacerbate these metabolic disturbances (Kane et al., 2004). Many APDs currently available in the United States have been associated with metabolic side effects to varying degrees (Kendall, 2011). Clozapine and olanzapine, though the most clinically effective APDs available, have also shown the greatest risk for inducing IR and significantly elevate risks for major cardiovascular events (e.g., acute coronary syndrome, ischemic stroke, and peripheral artery disease) by up to 2.8-fold (Szmulewicz et al., 2017). Indeed, 32% of patients taking olanzapine develop IR, in addition to gaining at least 15% of their baseline bodyweight (Citrome et al., 2011). Such APD-induced IR is a critical factor in the increased risk for coronary vascular disease, and thus is one of the principal causes of morbidity and premature mortality in this population (Hennekens et al., 2005; Laursen et al., 2012). Other APDs such as, ziprasidone and lurasidone have lesser cardiometabolic risks (Allison et al., 1999), though still elevate risk for other metabolic abnormalities such as, non-alcoholic fatty liver disease (Morlan-Coarasa et al., 2016).

Significantly, while much of the field's focus on metabolic side effects has been on newer medications (second-generation or atypical APDs), even first-generation APDs, such as, haloperidol or chlorpromazine, have been associated with weight gain and IR (Gordon et al., 1960; Fleischhacker et al., 2012). Consistent with this, in the large EUFEST trial (European First Episode Schizophrenia Trial), while olanzapine produced a 13.9 kg weight gain in subjects compared to baseline, haloperidol still caused a significant 7.3 kg weight gain as well (Kahn et al., 2008; Fleischhacker et al., 2012). Similarly, the “Evaluation of METabolic disordErs in schizOphRenic patients” (METEOR) study found no significant differences between patients prescribed first- or second-generation APDs in terms of the prevalence of glycemic disorders, dyslipidemia, and metabolic disorder (Falissard et al., 2011). These findings argue that most, if not all, APDs may cause or exacerbate metabolic symptoms and associated morbidity (Kahn et al., 2008). Data from the CATIE (Clinical Antipsychotic Trials of Intervention Effectiveness) study also showed substantial effects on metabolic in subjects taking APDs (McEvoy et al., 2005). When compared with a matched sample of people without psychiatric illness from the National Health and Nutrition Examination Survey (NHANES), people with schizophrenia treated with APDs had the highest metabolic risk of any patient group (McEvoy et al., 2005). These results reaffirm that, independent of medication effects, schizophrenia appears to confer an intrinsic risk of metabolic dysfunction, and APD treatment exacerbates this preexisting susceptibility to metabolic disease (Spelman et al., 2007). Therefore, the *combination* of inherent metabolic risks, both genetics and lifestyle, when combined with APD treatment, leads to the greatest possible metabolic risk category in medicine.

## Central nervous system mechanisms of intrinsic and APD-induced metabolic disturbances

Most studies examining either APD-induced metabolic side effects or intrinsic metabolic risk in schizophrenia have focused primarily on regions of the central nervous system (CNS) associated with metabolic control (e.g., hypothalamus). Consequently, numerous neurotransmitter and neuropeptide systems in the brain have been implicated in mediating these metabolic phenomena including the monoamines dopamine, serotonin, and histamine (Nasrallah, 2008).

### Dopamine

The single unifying property of all APDs is their ability to act on dopamine receptors including dopamine D_2_ and D_3_ receptors (D2R and D3R). Consequently, increasing evidence suggests that central D2R/D3R play important roles in mediating both APDs' therapeutic actions as well as their metabolic side effects (Beaulieu et al., 2005; Karam et al., 2010; Ballon et al., 2014). Moreover, network and pathway-based analyses also suggest that these receptors are jointly associated with both schizophrenia and T2D, further implicating these receptors as mediators of intrinsic metabolic risk in schizophrenia (Liu et al., 2013). Indeed, D2R is expressed in the pituitary gland in lactotroph cells that produce and release of prolactin, a powerful hormone regulator of systemic glucose homeostasis (Lopez Vicchi et al., 2016); D2R also regulates proliferation of the lactotrophs themselves (Ben-Jonathan and Hugo, 2015; Lopez Vicchi et al., 2016). Additionally, D2R is involved in central regulation of appetite via signaling through the striatal reward pathways. Mutations or polymorphisms of D2R associated with diminished D2R levels in the CNS have been implicated with increased feeding motivation, food intake and development of overweight states (Wang et al., 2001, 2002; Palmiter, 2007). Lastly, dopamine and D2R signaling in hypothalamic regions such as, the suprachiasmatic nucleus may mediate the circadian rhythms responsible for metabolic control including systemic insulin sensitivity (Landgraf et al., 2014, 2016; Barandas et al., 2015).

### Serotonin

In addition to dopamine, serotonin and serotonin receptors have been implicated in both intrinsic and APD-induced metabolic disturbances in schizophrenia (Kroeze et al., 2003; Tang et al., 2014). Single-nucleotide polymorphisms in 5HT2a and 5HT2c serotonin receptors are associated with several sequelae of metabolic dysfunction including obesity, glucose intolerance, and weight gain (Kring et al., 2009). Similarly, in rodent models, loss of function 5HT2c receptor mutations produce insulin-resistant and hyperphagic mice (Nonogaki et al., 1998). As the case with dopamine, these studies suggest that perturbation of brain serotonin signaling may play roles in both T2D and schizophrenia. Furthermore, CNS serotonin receptors are important targets for APDs, especially for atypical antipsychotics such as, clozapine and olanzapine (Reynolds and Kirk, 2010; Arranz et al., 2011). Though individual APDs have different respective binding affinities at the serotonin receptors, it has been suggested that APD actions at 5HT2a and 5HT2c receptors in the hypothalamus contribute significantly to iatrogenic weight gain (Ballon et al., 2014). In contrast, a recent study showed that polymorphisms in serotonin receptor genes HTR3A and HTR3B were not associated with predicting APD-induced weight gain (Zai et al., 2016).

### Histamine

Histaminergic neurons are localized to the posterior hypothalamus and project throughout the brain including striatum. Increasing evidence suggests that brain histaminergic signaling plays important roles in feeding behaviors in part through its modulatory actions on the reward circuitry (Bolam and Ellender, 2016). Studies have demonstrated that histamine has an anorectic effect on food intake via its actions on histamine H1 receptors (H1R; Ishizuka and Yamatodani, 2012). Indeed, intracerebroventricular infusion of histamine in rodents reduces food intake likely through its actions on H1R (Ishizuka et al., 2006). Consistent with these observations, APDs with the greatest antagonist H1R affinity, clozapine, and olanzapine, also stimulate hypothalamic AMP-activated protein kinase (AMPK) that may culminate in increased appetite and feeding (Kim et al., 2007). Hypothalamic histamine H1R is also associated with modulation of systemic energy balance (He et al., 2013). Consequently, blockade of these histamine receptors by APDs leads to augmented activation of downstream AMPK-carnitine palmitoyltransferase 1 signaling of the receptors which culminates in increased appetite (He et al., 2013). Further, long term histamine receptor blockade causes fat accumulation by decreasing lipolysis in adipose tissue (He et al., 2013). Nevertheless, the roles of histamine receptors, including H1 and H3 receptors, in mediating metabolic risk in schizophrenia remain controversial (Shams and Muller, 2014). Relatively few genetic polymorphisms have been identified for these receptors and the results have been equivocal. For example, recent work examining SNPs in the genes encoding H1 and H3 receptors (HRH1 and HRH3, respectively) did not yield significant results for either receptor in mediating APD-induced weight gain (Godlewska et al., 2013; Tiwari et al., 2016).

## APD-induced effects on appetite and energy consumption

Weight gain is fundamentally due to an energy imbalance where there is a surplus of energy intake over energy consumption. This leads to storage of the excess energy typically leading to increased weight (Muller and Kennedy, 2006). Most commonly, these increases in energy intake are due to increased caloric intake secondary to enhanced appetite. In addition to the above evidence for APD actions on the histamine system in causing appetite changes that lead to obesity (Deng, 2013), second-generation APDs also act on several neuropeptides that modulate appetite and food intake. For example, olanzapine and risperidone have both been shown to increase levels of appetite-stimulating neuropeptides including neuropeptide Y (NPY) and agouti-related peptide (AgRP), along with elevations in H1R expression to further potentiate these effects (Lian et al., 2015). Olanzapine may also increase appetite through actions on ghrelin, an orexigenic peptide, where ghrelin receptor signaling is enhanced by the drug (Tagami et al., 2016). Likewise, APDs may further increase appetite by decreasing levels of proopiomelanocortin (POMC), an appetite-inhibiting neuropeptide (Lian et al., 2015). Importantly, POMC and its precursor pre-POMC are differentially processed to yield several other metabolically-relevant derivative molecules including β-endorphin, melanocyte-stimulating hormones (MSHs), and adrenocorticotropic hormone (ACTH; Millington, 2007; Mountjoy, 2015; Anderson et al., 2016). Recent work suggests that one of these derivatives, α-MSH, may play a role in appetite regulation (Vehapoglu et al., 2016). Consequently, decreased levels of α-MSH, an anorexigenic hormone, in response to risperidone treatment likely further contribute to the increases in appetite observed clinically (Baltatzi et al., 2008; Yanik et al., 2013). Evidence also suggests that one of the MSH receptors, melanocortin 4 receptor (MC4R), is strongly linked with both weight regulation and APD-induced obesity (Zhang et al., 2016). Indeed, MC4R function is implicated in energy expenditure as well as regulation of food intake independently of changes in body weight (Xu et al., 2013; Mountjoy, 2015). Furthermore, a single nucleotide polymorphism (SNP) in MC4R, rs17782313, has been associated with overeating as well as APD-induced weight gain (Yilmaz et al., 2015; Macneil and Muller, 2016). Two additional SNPs, rs8087522, and rs1801133, have also been linked to APD-induced weight gain (Malhotra et al., 2012; Macneil and Muller, 2016), suggesting that the *MC4R* locus may be an attractive candidate for predicting APD-induced weight gain and metabolic disruption.

In terms of energy expenditure, APDs such as olanzapine reduce locomotor activity in rodent models, which further disrupts the balance between energy intake and consumption and leads to weight gain (van der Zwaal et al., 2014). Moreover, these olanzapine-induced decreases in locomotor activity occur at doses that do not affect eating behavior (Weston-Green et al., 2011). It has been suggested that APDs' sedative properties play an important role in causing this diminished energy consumption (van der Zwaal et al., 2014). Consistent with this, there is significant incidence of somnolence in people treated with many second-generation APDs (Gao et al., 2008). Furthermore, thermogenesis is another measure of body energy consumption and olanzapine administration was shown to cause reductions in body core temperature in animal model (van der Zwaal et al., 2012, 2014). Therefore, these drug-induced decreases in body core temperature suggest overall decreases in energy expenditure. Nevertheless, it is unclear to date if this is the case. Several trials have shown either no significant effect of APDs on resting energy expenditure (REE; Graham et al., 2005; Vidarsdottir et al., 2010), decreased REE (Sharpe et al., 2005, 2006; Nilsson et al., 2006) or even elevated REE (Fountaine et al., 2010). Intriguingly, unmedicated people with schizophrenia exhibited decreased REE (Nilsson et al., 2006). Overall, these studies suggest that the relationships between changes in thermogenesis and REE are likely complex and APD effects on body core temperature may cause compensatory changes in REE.

The ability of APDs to differentially target the various respective receptor signaling systems may have profound effects on both appetite and energy balance. Differences in the respective APDs' actions on both arms of energy balance regulation is likely responsible both for differences in the magnitude of significant metabolic side effects caused by these medications. However, this is complicated clinically by the observation that there is relatively high overall variability in the phenotypes of APD-induced metabolic dysfunction. Of the people who develop metabolic side effects, some gain weight with no IR; some develop IR without weight gain, and some experience both. Based on the discrepancy between weight gain and IR, an alternate treatment approach has been to focus on effects of APDs on appetite (Mizuno et al., 2014). Therapeutically, this has led to studies of appetite suppressants like topiramate and dextroamphetamine, to target CNS appetite centers. Nevertheless, appetite suppressants, including topiramate, have little to no effect in treating either intrinsic or APD-induced metabolic dysfunction, and cause significant cognitive and/or psychotic side effects that limit their overall safety (Narula et al., 2010; Muscatello et al., 2011; Mizuno et al., 2014). This highlights the conundrum concerning the roles of monoamine signaling in schizophrenia's intrinsic effects on metabolism and APD-induced metabolic effects: despite significant strides in understanding the physiology and pharmacology of monoamine signaling in the CNS, there has been a paucity of major mechanistic breakthroughs or new treatments that have mitigated these metabolic disturbances (Mizuno et al., 2014).

## Peripheral mechanisms of intrinsic and APD-induced metabolic disturbances

**Figure 1:**
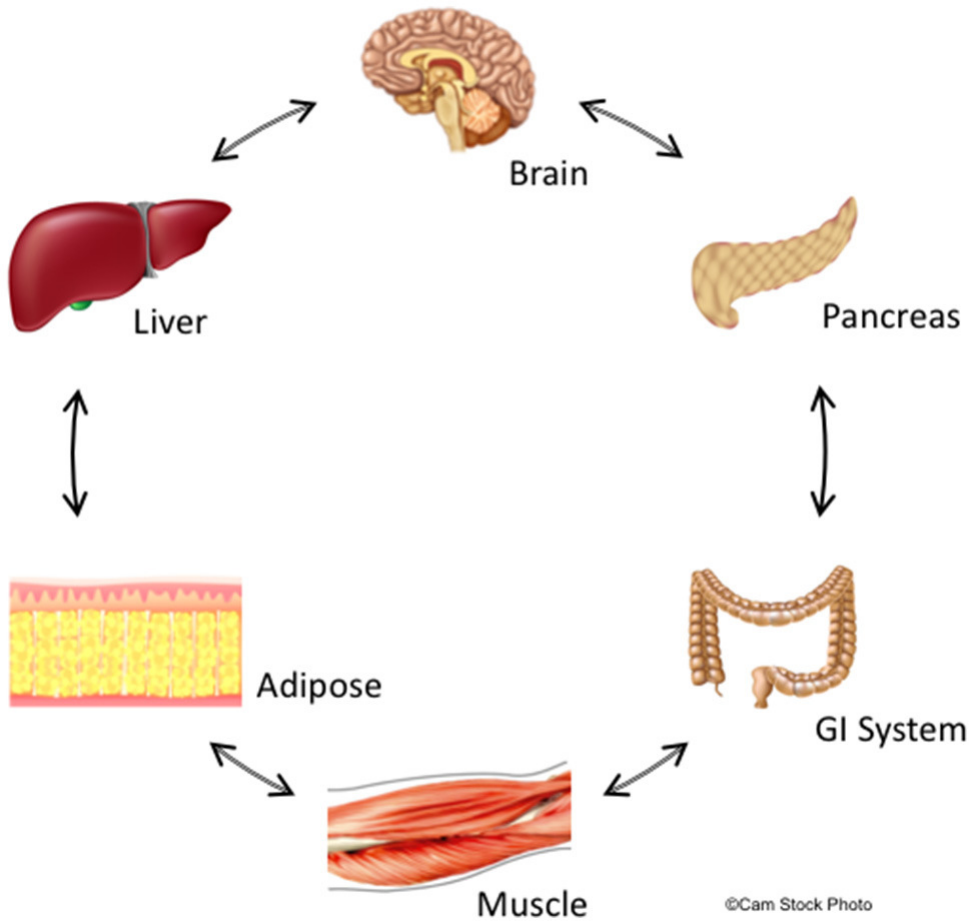
Summary of central and peripheral mechanisms contributing to deleterious effects of schizophrenia and APDs on glucose and lipid metabolism. The central nervous system,including metabolically-relevant areas in the brain such as hypothalamus, receive feedback from peripheral organs that regulate metabolism and appetite. Likewise, metabolic centers in the brain regulate metabolism throughout the body via actions on peripheral organs including GI tract, liver, pancreas, adipose tissue, and skeletal muscle. This feedback is bidirectional though non-sequential, and creates a delicate metabolic balance that is disturbed by biological changes intrinsic to schizophrenia and is further perturbed by APDs. We hypothesize that these central and peripheral target organs are linked through common molecular signaling networks involving dopaminergic, serotoninergic, histaminergic, and adipokine signaling. Since APDs act at receptors for these signaling systems, these drugs may have synergistic properties that significantly increase the risk of developing metabolic disturbances including insulin resistance and adiposity.

A potential explanation for the limited efficacy of drugs targeting APD-induced metabolic dysfunction is their focus primarily on CNS targets. Increasing evidence suggests that the same molecular targets of APDs, such as, histamine, serotonin, and dopamine receptors, also exist in peripheral organs critical for metabolic control, including the pancreas, adipose tissue, and skeletal muscle (Garcia-Tornadu et al., 2010; Rubi and Maechler, 2010; Ballon et al., 2014; see Figure 1). These peripheral targets, together with the CNS, jointly regulate both body weight and glucose/insulin homeostasis, key factors in APD-induced metabolic dysfunction (Ballon et al., 2014). For example, insulin-secreting pancreatic beta cells not only produce their own dopamine, but also express D2R, an important APD target (Rubi et al., 2005; Simpson et al., 2012). There is further precedent for CNS neurotransmitter action in the periphery, including in the gastrointestinal tract and adipose tissue (Gershon, 2013). Moreover, there is crosstalk between the CNS and these peripheral organs since hypothalamic and brain stem neurons have been shown to influence beta cell, hepatocyte and adipocyte metabolism (Elmquist et al., 2005) and vice versa.

**Figure 2:**
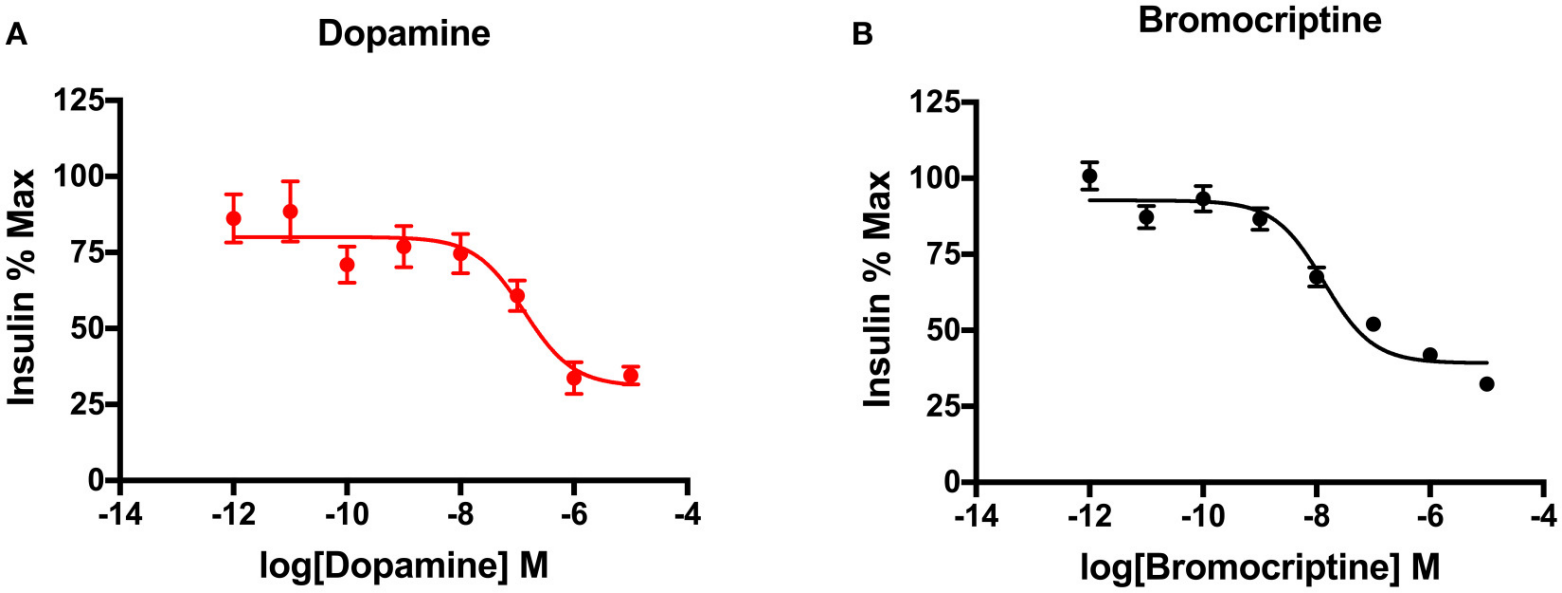
Dopaminergic modulation of insulin secretion in pancreatic beta cells. **(A)** Treatment of INS-IE cells, an established rat pancreatic beta cell-derived cell line, with dopamine potently inhibited glucose-stimulated insulin secretion in a dose-dependent manner (pIC_50_ = 7.83). **(B)** Agonism of dopamine D_2_ and D_3_ receptors by bromocriptine produced a decrease in glucose-stimulated insulin secretion comparable to dopamine (pIC_50_ = 6.87), suggesting that these receptors are important in mediating this effect in beta cells. Insulin secretion was measured via homogenous time-resolved fluorescence (HTRF) as described earlier (Farino et al., 2016). All experiments were performed in triplicate on *n* ≥ 3 separate experimental days. Data are represented as % maximal insulin secretion based on mean HTRF values ± SEM.

Glucose and insulin-sensitive tissues in the periphery, including pancreatic beta cells and adipose cells, express D_2_-like receptors (D2R, D3R, and D4R), which are key targets of APDs (Wilson et al., 1998). In addition, insulin-secreting pancreatic beta cells also express the CNS-specific isoform of the vesicular monoamine transporter, VMAT2, which is responsible for vesicular dopamine loading and storage (Anlauf et al., 2003). We have shown that dopamine acts as an autocrine or paracrine negative regulator of glucose-stimulated insulin secretion (GSIS) to dampen further insulin release (Figure 2A; Simpson et al., 2012). Moreover, we have also demonstrated that the addition of sulpiride, one of the most D2R/D3R-selective APDs (Newman et al., 2012), increases GSIS by 40% in pancreatic beta cells. Importantly, this APD-induced increase in insulin secretion is consistent with the chronic hyperinsulinemia found in APD-induced IR clinically (Henderson et al., 2005). Conversely, D2R and D3R agonism may have therapeutic effects on IR. Indeed, a quick-release formulation of the D2R/D3R agonist bromocriptine is FDA-approved to treat T2D (Mikhail, 2011; Lamos et al., 2016). Although the majority of work studying bromocriptine's metabolic effects has focused on its actions in the CNS, there is robust evidence that bromocriptine also targets the same peripheral dopamine signaling pathways altered by APDs, as described above (Holt et al., 2010; Farino et al., 2016; Lamos et al., 2016). Bromocriptine lowers both elevated glucose and insulin levels in humans (Liang et al., 1998; Holt et al., 2010). Based on this evidence, bromocriptine may be effective in treating APD-associated IR by targeting peripheral dopamine pathways disrupted by APDs. Consistent with this, data from small studies is validating this hypothesis, having demonstrated reestablishment of euglycemia in the context of APD treatment (Naguy and Al-Tajali, 2016). On a cellular level, we have now demonstrated that bromocriptine treatment diminishes GSIS comparably to dopamine in a dose-dependent manner in a beta cell-derived cell line (Figure 2B). These data suggest that dopamine D_2_-like receptors may play an important role in the periphery to modulate secreted insulin. Furthermore, the disruption of this regulatory signaling either through genetic changes intrinsic to the disease process in schizophrenia or via drug action by APDs may lead to many of the metabolic disturbances observed clinically.

## Existing treatment strategies

Given the importance of peripheral metabolic targets, initial attempts at treating APD-associated IR focused on current T2D medications already known to act in the periphery (Mizuno et al., 2014). The widely-used class of T2D drugs, thiazolidinediones, increase insulin sensitivity (IS) in T2D by acting on peripheral targets including adipose tissue, skeletal muscle, and liver to increase glucose utilization and decrease glucose production (Hauner, 2002). Besides T2D, these drugs have been applied to treatment of metabolic dysfunction associated with psychiatric disorders. However, when rosiglitazone was studied in people with schizophrenia on clozapine, it was ineffective (Henderson et al., 2009). Given these results with thiazolidinediones, further trials looked to other classes of insulin sensitizing drugs. Metformin, a biguanide, improves blood glucose levels in T2D through decreased hepatic glucose production and increased peripheral glucose utilization (Zhou et al., 2001). Though metformin modestly decreases APD-induced weight gain, like the thiazolidinediones, it has also failed to meet expectations for improving APD-induced IR (Jarskog et al., 2013; Rado and Von Ammon Cavanaugh, 2016). Based on the relative failure for using these otherwise well-established mechanistic strategies to treat IR in the setting of ongoing APD treatment, a new therapeutic strategy is clearly needed. To date, such attempts have been hampered by the relative paucity of knowledge regarding the biological modulators of metabolic dysfunction both in the cases of intrinsic and APD-induced metabolic dysfunction. Below we will review the roles of several such modulators.

## Adiponectin and metabolic dysfunction

Adiponectin is an adipokine hormone, produced primarily by fat cells in adipose tissue that makes tissues more sensitive to insulin (Diez and Iglesias, 2003). Adiponectin levels are inversely related to obesity (Di Chiara et al., 2012). Further, low levels of circulating adiponectin are associated with IR and may provide a link between obesity and overall metabolic syndrome (Di Chiara et al., 2012). Increasing adiponectin leads to improvements in insulin sensitivity (IS) through enhanced tissue fat oxidation in adipose tissue, lowering circulating fatty acid levels and reducing intracellular triglyceride contents in liver and muscle (Diez and Iglesias, 2003). It further lowers inflammation and thus reduces risk of atherosclerosis, by suppressing the expression of adhesion molecules in vascular endothelial cells and cytokine production from macrophages. By suppressing the initial inflammatory process associated with early atherosclerosis, increasing levels of adiponectin lower the risk for cardiovascular sequelae of metabolic dysfunction (Diez and Iglesias, 2003). The combination of improving IS while lowering inflammation has generated strong interest in metabolic treatments that increase adiponectin.

The relationship between drug-naïve schizophrenia and adiponectin is unclear. An initial study found lower serum adiponectin levels in normal-weight, first-episode, drug-naïve, people with schizophrenia (Song et al., 2013). A recent meta-analysis was unable to confirm the finding, though did show a trend level of significance (*p* = 0.09) for decreased adiponectin in drug-naïve individuals with schizophrenia (Bartoli et al., 2015). The result was notable for significant heterogeneity within the sample, which indicates that there is a subset of people with schizophrenia who have abnormally low baseline adiponectin levels and may be at greater risk for APD-induced metabolic side effects. The introduction of APDs has been consistently shown to lower adiponectin levels (Bai et al., 2009; Tanyanskiy et al., 2015). The drugs most associated with decreased adiponectin are also the drugs most associated with metabolic side effects, clozapine (*p* < 0.001) and olanzapine (*p* = 0.04; Bartoli et al., 2015). Furthermore, dopamine receptors in adipocytes appear to regulate adiponectin levels, which suggests a putative link between the D2R/D3R polymorphisms implicated in the pathogenesis of schizophrenia and adiponectin's role in mediating intrinsic metabolic disturbances in people with the illness. Likewise, D2R/D3R antagonism ubiquitous to APDs may also connect adiponectin to APD-induced metabolic dysfunction (Borcherding et al., 2011).

Given the above evidence that the machinery for monoamine synthesis and dopamine receptor signaling is present in insulin-sensitive peripheral organs, it suggests that: (1) APDs target the same monoamine receptors in the periphery as they do in the CNS, and (2) monoamines are not only involved in APD-induced IR, but in overall insulin homeostasis (Bailey, 2000). Increasing evidence points to a potentially important contribution by monoamines to APD-induced metabolic dysfunction through direct action of APDs on peripheral monoaminergic targets including those found in insulin-secreting pancreatic beta cells (Kalra et al., 2011). This may therefore be consistent with D2R/D3R agonist bromocriptine's efficacy in the treatment of T2D based on findings that it reduces plasma glucose, triglyceride, and free fatty acid levels, and significantly decreases hemoglobin A1C levels as compared to placebo (Holt et al., 2010; Valiquette, 2011). (2) Robust metabolic findings have also been seen in obese, non-diabetic people comparable at least by metabolic markers to people with schizophrenia (Kamath et al., 1997; Kok et al., 2006).

In sum, it is increasingly evident that effects on metabolism caused by APD treatment and/or those intrinsic to schizophrenia are due to convergent effects on signaling through monoamines or neuromodulators in both the CNS and periphery. Metabolically-relevant tissues in the CNS and periphery share many of the same receptor signaling systems including the dopamine, serotonin, and histamine systems. Moreover, changes in central brain metabolic regulation propagate to target organs in the periphery including the gastrointestinal system (GI), pancreas, muscle, liver, and adipose tissues and these tissues respond in a reciprocal manner. Because APDs act concurrently on multiple receptor systems in all these tissues (e.g., dopamine, serotonin, histamine), the effects of these drugs' disruptions on metabolic regulation and appetite are likely cumulative and lead to the metabolic imbalances observed clinically (Figure 1).

## Genes implicated in both schizophrenia and metabolism

To date, there is little definitive evidence of genes associated with schizophrenia having a causative role in the development of metabolic sequelae associated with either APD-induced metabolic disturbances or with the illness' intrinsic metabolic risk. Nevertheless, two genes associated with schizophrenia, *AKT1* and neuregulin 1 (*NRG1*), may also play roles in systemic metabolic regulation. This suggests that mutations or polymorphisms in these genes may have roles not only in neuronal function within the CNS associated with schizophrenia's neuropsychiatric symptoms, but that these disruptions of function may also affect more global metabolic functions in other pathways including within insulin signaling in organs such as, the pancreas and liver.

### AKT1

The gene encoding the serine-threonine kinase AKT1 has long been implicated in schizophrenia across multiple studies (Xu et al., 2007; Thiselton et al., 2008; Karam et al., 2010). AKT1 protein levels were diminished both in peripheral lymphocytes and in the brains of people with schizophrenia (Emamian et al., 2004; Arnold et al., 2005). Moreover, in rodent models, treatment with the APD haloperidol changed the phosphorylation responsible for regulating this kinase's ability to signal through its actions on another kinase, glycogen synthase kinase 3β (GSK3β) which was attributed as a potential mechanism for haloperidol's therapeutic efficacy (Emamian et al., 2004; Beaulieu et al., 2005). AKT1 is also especially relevant to intrinsic metabolic risk in schizophrenia since it is an important regulator of insulin signaling. Insulin receptor activity triggers phosphatidylinositol 3 kinase (PI3K)-dependent recruitment of AKT1 to the cell surface where it is subsequently activated (Guo, 2014). Conversely, loss of function AKT1 mutations are implicated in IR, suggesting a key role for the enzyme in metabolic control (Bernal-Mizrachi et al., 2004). Consistent with this, AKT phosphorylates downstream transcriptional activators including Foxo1 and SREBP1c that regulate glucose transport and insulin sensitivity in tissues throughout the body (Gonzalez et al., 2011; Guo, 2014). AKT1 signaling has also been implicated in APD-induced metabolic dysfunction, potentially through its effects on Wnt and beta catenin-mediated transcriptional changes (Freyberg et al., 2010). Significantly, the activity of AKT1 is modulated by D2R signaling where D2R activation leads to AKT1 inactivation (Beaulieu et al., 2007; Beaulieu and Gainetdinov, 2011). By this logic, it would be expected that D2R blockade by APDs would lead to increased levels of active AKT1 and therefore more effective insulin action rather than increased IR. A potential explanation of this apparent discrepancy is that AKT1 protein levels are diminished in people with schizophrenia (Emamian et al., 2004). Therefore, APD blockade of D2R receptors may be ineffective in enhancing insulin signaling since there is already a deficit of AKT1 signaling downstream of D2R and may even lead to changes in AKT-mediated regulation of metabolism (Freyberg et al., 2010).

### NRG1

*NRG1* encodes a growth factor that belongs to the larger family of epidermal growth factor (EGF) family critical for development of multiple organ systems including the nervous system, liver, heart, and skeletal muscle (Britsch, 2007; Guma et al., 2010; Mei and Nave, 2014). Of the four neuregulin isoforms described, polymorphisms in the *NRG1* gene have been repeatedly associated with schizophrenia (Banerjee et al., 2010; Karam et al., 2010; Caillaud et al., 2016; Mostaid et al., 2017). Moreover, *NRG1* SNPs were recently associated as predictors of APD clinical response (Li et al., 2017). Though data from computational pathway analyses have implicated interactions between NRG1 and Src signaling pathways which play roles in both schizophrenia and T2D (Liu et al., 2013), to date, the links between NRG1 and the metabolic disturbances intrinsic to schizophrenia are not well-established. However, in rodent models, NRG1 function has been shown to be important in glucose metabolism and insulin sensitivity both in liver and muscle (Caillaud et al., 2016; Lopez-Soldado et al., 2016). This may be attributable to NRG1's role in regulating hepatic glucose utilization and gluconeogenesis (Arai et al., 2017). Taken together, these data suggest that NRG1 may play a modulatory role in both in the CNS and systemically and disruption of its signaling either intrinsic to the disease processes in schizophrenia and/or with APDs may lead to the neuropsychiatric and metabolic dysfunction observed clinically.

## Conclusions

We suggest a model, based on the above evidence, in which the disease processes underlying schizophrenia also confer a significant intrinsic risk for development of metabolic disturbances including IR and T2D. Consequently, these processes not only affect regions of the CNS implicated in the neuropsychiatric symptoms classically associated with the disorder including cognition, executive function and sensory perception, but likely target metabolically-relevant areas as well. Changes in neurotransmission within CNS regions such as, the hypothalamus in schizophrenia may also feedback upon metabolic signaling in the periphery including the endocrine pancreas, liver, and adipose tissue (Figure 1). Moreover, because these central and peripheral targets likely rely on conserved signaling pathways and molecules (e.g., dopamine, AKT1, NRG1), disruption or changes in these pathways in one organ may also be found in others and consequently may explain the reciprocal connections between multiple systems. Furthermore, APDs may further alter metabolism through their actions at these same CNS and peripheral sites of action. This results in APD-induced exacerbation of the preexisting metabolic risks intrinsic to schizophrenia. Along with additional lifestyle and environmental factors, the combination of APD-induced and intrinsic risk factors leads to the serious metabolic dysfunctions described clinically (Karam et al., 2010; Ballon et al., 2014). Therefore, improving our understanding of both the processes responsible for metabolic regulation and schizophrenia may elucidate the common mechanisms between both and ultimately inform new drug development strategies.

## Author contributions

ZF and JB: Conception and design, manuscript writing, editing and figure design. DA: Experimental work and conception and figure design. RS: Manuscript writing, editing, synthesis of previous literature and figure design.

### Conflict of interest statement

The authors declare that the research was conducted in the absence of any commercial or financial relationships that could be construed as a potential conflict of interest.
